# A research-based gene panel to investigate breast, ovarian and prostate cancer genetic risk

**DOI:** 10.1371/journal.pone.0220929

**Published:** 2019-08-15

**Authors:** Madison R. Bishop, Anna L. W. Huskey, John Hetzel, Nancy D. Merner

**Affiliations:** 1 Auburn University, Harrison School of Pharmacy, Department of Drug Discovery and Development, Auburn, Alabama, United States of America; 2 Auburn University, College of Veterinary Medicine, Department of Pathobiology, Auburn, Alabama, United States of America; CNR, ITALY

## Abstract

There is a need to investigate and better understand the inherited risk of cancer to ensure that clinical applications provide more accurate assessments and management strategies. Developing research-based next-generation sequencing gene panels that not only target (present-day) clinically actionable susceptibility genes but also genes that currently lack sufficient evidence for risk as well as candidate genes, such as those in DNA repair pathways, can help aid this effort. Therefore, gene panel B.O.P. (**B**reast, **O**varian, and **P**rostate) was developed to evaluate the genetic risk of breast, ovarian and/or prostate cancer, and this manuscript serves as an introduction to B.O.P. and highlights its initial analytical validity assessment. B.O.P targets 87 genes that have been suggested, predicted, or clinically proven to be associated with breast, ovarian, and/or prostate cancer risk using Agilent Technologies Haloplex probes. The probes were designed for 100 base pair reads on an Illumina platform and target both coding and non-coding exons as well as 10 intronic base pairs flanking the intron-exon boundaries. The initial B.O.P screening involved 43 individuals from the Alabama Hereditary Cancer Cohort, and an average sequencing depth of 809X was obtained. Upon variant filtering and validation, true positives had an average sequencing depth of 659X and allele balance of 0.51. The average false positive sequencing depth was 34X and allele balance was 0.33. Although low sequencing depth was not always indicative of a false positive, high sequencing depths (>100X) signified a true positive. Furthermore, sensitivity and specificity of *BRCA1/2* were calculated to be 100% and 92.3%, respectively. Overall, this screening enabled the establishment of criteria to alleviate future validation efforts and strongly supports the use of B.O.P. to further elucidate hereditary cancer susceptibility. Ultimately, continued B.O.P. screening will provide insights toward the genetic risk of and overlap between breast, ovarian, and/or prostate cancer.

## Introduction

Gene panels enable the simultaneous screening of a number of genes. Panels are typically customized for specific screening purposes; thus, the genes (and/or specific gene regions) on such panels are unique to the screening goals. In recent years, with technological sequencing advances, panel-based screening has become extremely efficient and cost-effective. These advancements involve the targeted enrichment of selected genes followed by massively parallel sequencing, which is also known as next-generation sequencing (NGS) [1, 2].

NGS gene panels have been implemented into clinical practice to assess inherited risk of cancer [1–3]. These panels include clinically valid genes for which clinical management guidelines have been established, such as genetic risk assessment criteria and mutation-positive management strategies. In the U.S., the National Comprehensive Cancer Network (NCCN) provides such guidelines to maximize clinical utility [4]. The American College of Medical Genetics and Genomics (ACMG) has established clinical laboratory standards for NGS gene panels but, ultimately, these panels are regulated by the Clinical Laboratory Improvement Amendments (CLIA), a federal program that certifies and oversees clinical laboratory testing [5, 6]. CLIA primarily assesses analytical validity–the accuracy of mutation detection–in order to maintain quality standards and ensure the effectiveness of each laboratory test [6].

CLIA does not regulate research-based genetic testing, but similar analytical assessments can be carried out to ensure accurate mutation detection in a research setting. We have developed a research-based gene panel, B.O.P. (**B**reast, **O**varian, and **P**rostate), to assess inherited risk of hereditary breast cancer (BC) and associated cancers. B.O.P. is an exploratory gene panel. In addition to targeting clinically valid genes that have NCCN management strategies, it also targets genes that have been suggested to be associated with an increased risk but currently lack sufficient evidence, as well as candidate genes, such as those in DNA repair pathways. Therefore, the ultimate goal in utilizing this panel is to better elucidate risk. Regarding hereditary BC, NCCN clinically valid genes only account for a minority of the associated genes reported in the literature [1, 7, 8]. Furthermore, NCCN risk management strategies have primarily been developed for overtly pathogenic, truncation mutations in clinically valid genes–resulting in the detection of many variants of unknown significance (VUS), and clinically valid mutations explain less than 30% of hereditary BC cases. Additional exploration is critical to fill these knowledge gaps, and B.O.P. can aid in this investigation. However, prior to using B.O.P. as a way to increase knowledge in these areas, it must be evaluated for its ability to accurately detect variants. The purpose of this manuscript is to introduce B.O.P., present the analytical assessment of 10 NCCN regulated genes (in order to ensure the accurate detection of clinically relevant variants), and discuss the future potential of the panel.

## Materials and methods

### Ethical compliance and informed consent

All procedures performed in studies involving human participants were in accordance with the ethical standards of Auburn University and with the 1964 Helsinki declaration and its later amendments or comparable ethical standards. Specifically, this research was reviewed and approved by the Auburn University Institutional Review Board for the recruitment, enrollment and biobanking of the Alabama Hereditary Cancer Cohort (AHCC; IRB protocols 14–232, 14–335, and 15–111) [9]. Informed consent was obtained in writing from all individual participants included in the study.

### Panel design

B.O.P. targets ~500 kilobases (Kb) of DNA including 87 genes that are suggested, predicted, or clinically proven to be associated with BC, OvC, and/or prostate cancer (PC) risk (S1 Table). Agilent Technologies Haloplex probes were designed using Agilent Technologies SureDesign software (https://earray.chem.agilent.com/suredesign/). The “Advanced HaloPlex” design allowed for the selection of the desired genes of which the targeted regions included both coding and non-coding exons as well as 10 intronic base pairs flanking the intron-exon boundaries. The probe set was designed for 100 base pair reads on an Illumina platform. Overall, probes were predicted to cover 98.93% of the targeted genes/regions (Table 1).

**Table 1:** Ten clinically relevant genes on the B.O.P. panel assessed for analytical validity.

Gene or targeted regions		Accession number	# of targeted regions	Size (bp)	Predicted target 1X coverage* (%)	Average sequencing depth (X)	Interquartile range (X)			% bases covered greater than or equal to:								
							First quartile	Median	Third quartile	1X	10X	20X	50X	100X	250X	500X	1000X	10000X
Genes investigated for analytical validation	*ATM*	NM_000051	65	15545	98.0	781	336	659	1068	97.5	95.7	93.6	87.4	78.3	60.0	41.0	20.8	0.8
	*BRCA1*	NM_007300	24	7750	98.5	1017	397	787	1271	98.1	97.1	95.6	90.5	81.9	65.2	47.4	27.2	1.1
	*BRCA2*	NM_000059	28	12078	99.1	960	445	803	1225	98.7	97.8	96.5	91.6	83.5	66.6	48.1	25.7	1.1
	*CDH1*	NM_004360	16	5269	98.9	934	418	834	1242	98.5	97.1	95.5	91.1	83.4	66.3	48.3	26.6	1.0
	*CHEK2*	NM_001005735	23	4605	96.5	726	279	588	1023	95.8	93.4	91.3	84.5	74.7	56.6	38.5	18.9	0.7
	*NBN*	NM_002485	22	6681	98.0	696	312	610	959	97.6	96.0	93.8	86.6	76.8	57.9	38.0	17.9	0.6
	*PALB2*	NM_024675	13	4318	100.0	1001	557	876	1261	99.9	99.0	98.0	94.2	86.8	70.9	52.3	28.3	1.1
	*PTEN*	NM_000314	10	10248	98.0	597	221	468	853	96.9	93.2	90.0	81.3	70.6	50.8	31.3	14.1	0.5
	*STK11*	NM_000455	10	3476	100.0	505	175	387	774	93.2	89.0	85.9	77.6	67.5	46.5	28.3	11.5	0.3
	*TP53*	NM_000546	14	4216	99.0	788	316	676	1039	97.4	94.6	92.6	87.3	77.7	59.3	41.7	21.4	0.8
All targeted B.O.P. regions			1417	499,521	** **98.9	809	359	687	1092	98.2	96.6	94.7	88.8	79.9	61.9	43.0	21.6	0.8

*Based on design report by Agilent Technologies

### Capture and sequencing

**Fig 1:**
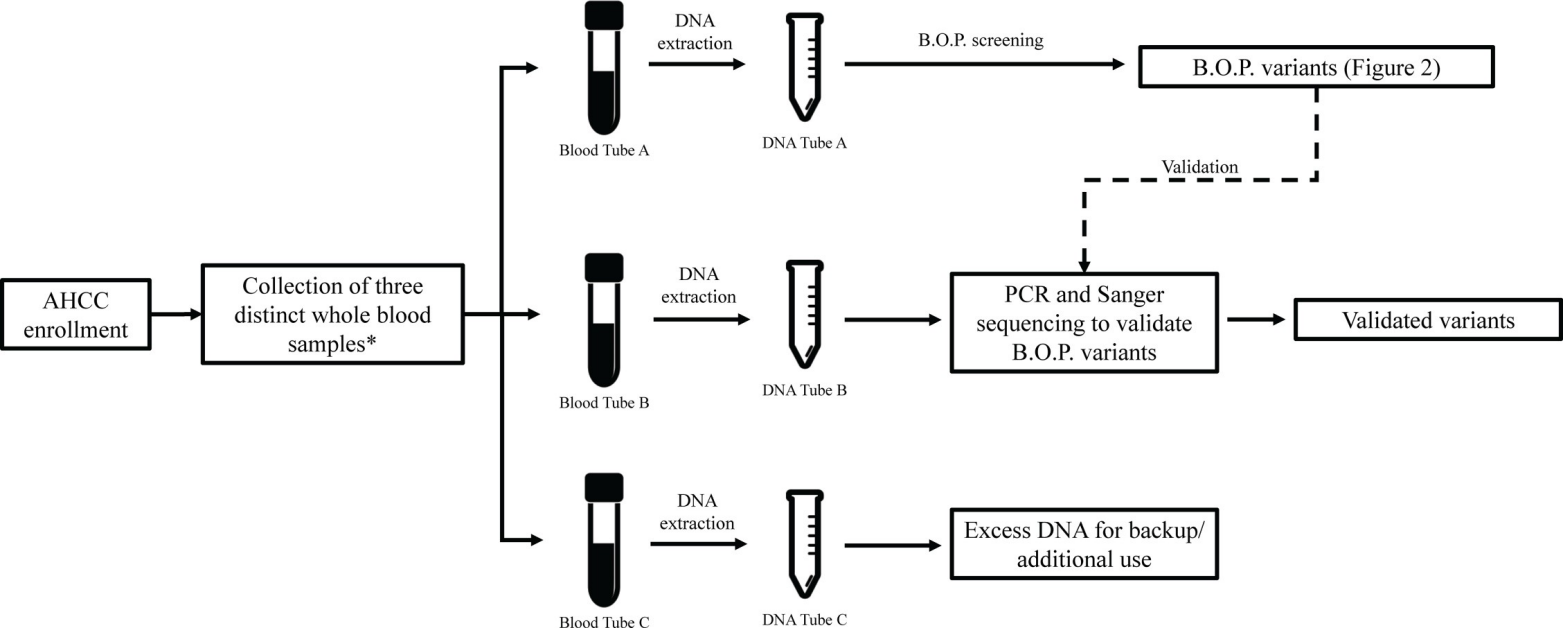
Screening process for individuals in the AHCC. *In situations where blood is unattainable, another set of three distinct biological samples (i.e. saliva) is collected.

**Fig 2:**
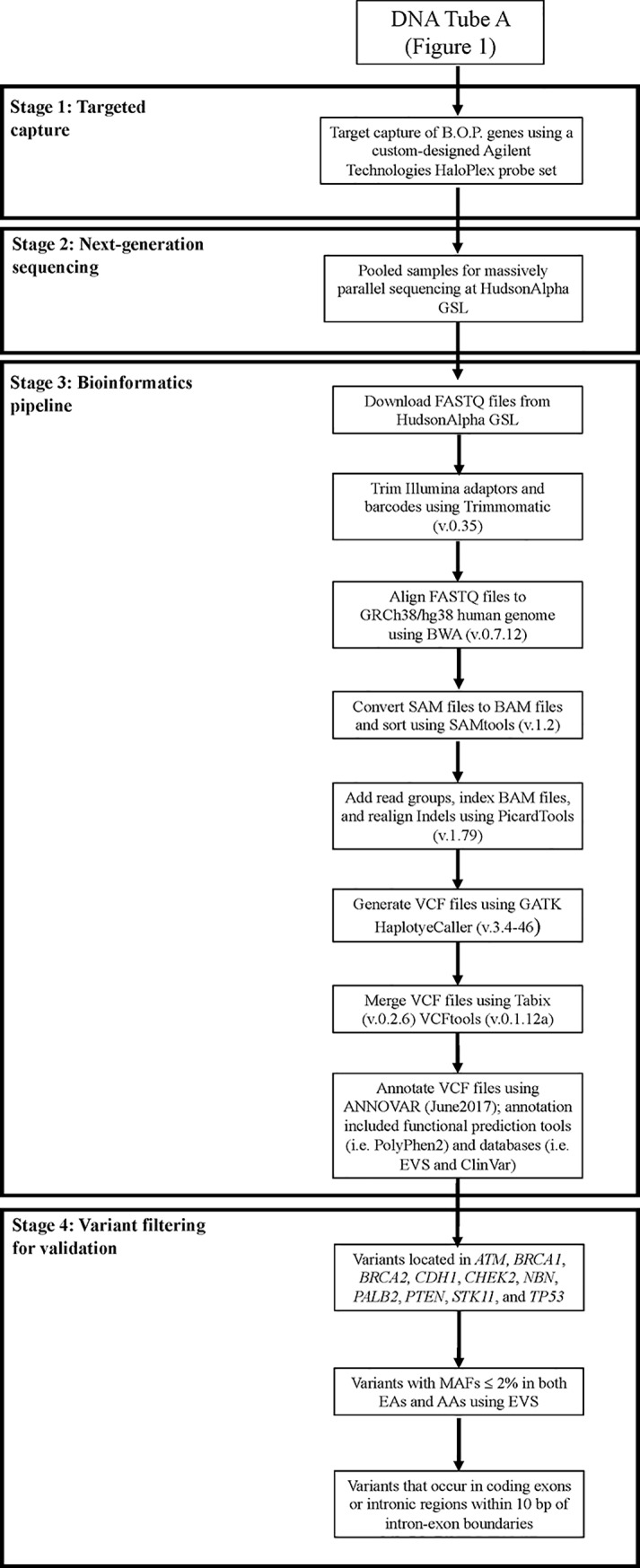
Pipeline for B.O.P. panel screening: Targeted capture, next-generation sequencing, bioinformatics pipeline, and variant filtering.

The genomic DNA of 43 cancer–affected individuals (23 African American [AA] and 20 European American [EA]) from the AHCC [9] was selected to undergo the first B.O.P. screening (Figs 1 and 2). Two study participants (1CAD-a and 1CAD-f) were knowingly related (first cousins). The HaloPlex HS Target Enrichment System For Illumina Sequencing Protocol (Version C0, December 2015) was followed for the targeted-capture, allowing each of the 43 samples to be uniquely barcoded/indexed, individually captured, and pooled in equimolar amounts for Illumina paired-end sequencing. One pooled sample with a final concentration of 24.13 nanomoles/liter (and DNA fragments ranging from 175 to 625 base pairs) was sent for sequencing on one lane of a flow cell on an Illumina HiSeq 2500 at the Genomic Services Laboratory (GSL) at HudsonAlpha Institute for Biotechnology. The final DNA quality/quantity of the pooled sample was assessed using the High Sensitivity DNA kit using the ABI 2100 Bioanalyzer.

### Bioinformatics analyses

The sequencing data generated for each indexed sample (43 forward and 43 reverse FASTQ files) were downloaded using the GSL’s wget downloader (Fig 2). Trimmomatic (v.0.35) was used to trim the unique barcodes and Illumina adaptors. After generating trimmed FASTQ files, FastQC (v.0.10.1) was used to ensure that the repeated sequences had been trimmed from the sequences. The trimmed, paired forward and reverse FASTQ files were then aligned to the soft-masked human reference genome (GRCh38/hg38) using Burrows-wheeler Aligner (BWA v.0.7.12), generating SAM (Sequence Alignment/Mapping) files, which were then compressed into BAM (binary SAM) files and sorted using SAMtools (v.1.2). PicardTools (v.1.79) was used to add read groups and then index the sorted, compressed BAM files along with realigning insertions and deletions (indels). As previously suggested for Haloplex, duplicates were not marked or removed [10]. Variants in B.O.P targeted-regions were called from the sorted BAM files using the HaplotypeCaller tool in the Genome Analysis Tool Kit (GATK; v.3.4–46); the generated VCF (Variant Calling Format) files were then merged using Tabix (v.0.2.6) and VCFTools (v.0.1.12a). Variants in the merged VCF files were annotated using ANNOVAR (June2017). Overall, this pipeline was adapted from the GATK Best Practice Pipeline [11]. Lastly, Samtools flagstat (v.1.2) was used to gather metrics of the analyzed reads, and the DepthOfCoverage tool within the GATK (v.3.4–46) was used to calculate the depth of coverage for the targeted regions.

### Analytical assessment

Ten of the 87 genes were selected for our initial analytical validation efforts (Table 1). The selected genes represented clinically actionable BC susceptibility genes in order to facilitate analytical validity calculations, as well as to begin to determine the landscape of potential clinically significant variants in the AHCC. Using Exome Variant Server (EVS) as a control repository, B.O.P. variants in those 10 genes were filtered for minor allele frequencies (MAFs) of less than or equal to 2% in both EAs and AAs [12]. Variants were further filtered; all coding variants as well as intronic variants that were located within 10 base pairs of an intron-exon boundary were carried through for validation using polymerase chain reactions (PCR) and Sanger sequencing (Fig 1). Primer sequences and amplification conditions are available upon request. P values and Odds ratios (ORs) were calculated using Fisher exact test in R (v 3.5.1), which were not adjusted for multiple testing.

Upon consent and enrollment into the AHCC, study participants provided information about previous clinical genetic screening [9]. Thus, for genes with clinical screening results provided by the 43 participants involved in this initial B.O.P. screening, sensitivity and specificity were calculated. Sensitivity was defined as the total number of true positives (TPs) divided by the sum of the total number of TPs and false negatives (FNs; TPs/ [TPs + FNs]). TPs were defined as (i) variants that had been previously identified through clinical gene screening, initially confirmed in the research laboratory by PCR and Sanger sequencing, and, subsequently, detected upon B.O.P. screening, or, (ii) in the case of no clinical screening results, as variants detected upon B.O.P. screening and then validated through PCR and Sanger sequencing (Figs 1 and 2). FNs were variants that had been previously identified through clinical gene screening and confirmed in the research laboratory by PCR and Sanger sequencing but not detected through B.O.P. screening. Specificity was defined as the ratio of the total number of true negatives (TNs) over the sum the total number of TNs and false positives (FPs; TNs / [TNs + FPs]). TNs were defined as individuals who did not have a pathogenic variant detected through clinical gene screening as well as B.O.P. screening. FPs were defined as variants detected through B.O.P. screening but were not validated upon subsequent PCR and Sanger sequencing (Figs 1 and 2). Lastly, the false discovery rate (FDR; FP / [TP + FP]) was calculated for the 10 genes individually. FDRs were calculated in two ways, (i) including all B.O.P. called variants, and (ii) excluding B.O.P. variants with an allele balance less than or equal to 0.20.

## Results

The number of reads that passed QC assessment per individual averaged 11.3 million (M), with 98.6% of those reads mapping to the human genome. On average, 50.9% of the reads mapped to B.O.P. targeted regions (S2 Table). The average sequencing depth for all targeted base pairs was 809X; however, sequencing depth was not uniform with a large interquartile range (Table 1). A probe design report provided by Agilent Technologies predicted that 98.9% of the targeted base pairs would be covered at least 1X, which was similar to the actual coverage of 98.2%; thus, 1.8% of the targeted base pairs were not covered at all (Table 1). The 10 assessed genes had average sequencing depths that ranged from 505X-1017X (Table 1). Furthermore, assessment of the 225 different regions that targeted those 10 genes revealed that the majority had average sequencing depths between 800-899X but ranged from 68X-2053X (Table 2; S3 Table). Although rare, regions with average sequencing depths less than 100X missed on average 24.3% of the targeted base pairs and only covered 52.2%, 34.6%, and 21.3% of targeted base pairs at or greater than 20X, 50X, and 100X, respectively (Table 2; S3 Table). Regions with the highest average sequencing depth, 1500X or greater, had over 99% and 96.5% of the targeted base pairs covered at least 50X and 100X, respectively. However, 28.0% of the 225 regions-of-focus did not, on average, obtain 100% coverage at 1X, which included regions with average sequencing depths ranging from 68X-1354X (Table 2; S3 Table).

**Table 2:** Average sequencing depth analyses of the 225 regions targeting the ten B.O.P. genes being assessed.

Average region sequencing depth (X)	# of regions	Average % bases covered greater than or equal to:								
		1X	10X	20X	50X	100X	250X	500X	1000X	10000X
<100	2	75.7	63.2	52.2	34.6	21.3	7.3	1.8	0.9	0.0
100–199	4	84.4	75.9	69.1	54.8	39.5	15.6	5.0	2.3	0.0
200–299	7	91.5	88.8	82.6	70.1	54.9	26.1	8.1	2.7	0.0
300–399	12	97.0	93.1	88.2	77.9	64.5	38.5	16.5	5.0	0.0
400–499	22	97.8	95.6	92.8	83.2	71.8	47.9	24.8	7.0	0.1
500–599	21	98.4	96.9	94.8	87.5	76.0	56.7	32.9	11.1	0.2
600–699	27	99.3	98.2	96.2	89.6	79.2	59.3	37.9	14.9	0.5
700–799	28	99.1	97.8	96.6	91.1	81.6	64.5	42.7	19.1	0.7
800–899	29	99.6	98.9	98.0	93.8	85.2	68.8	49.4	24.1	0.9
900–999	13	100.0	99.7	99.2	96.3	88.6	73.2	54.0	27.8	1.2
1000–1099	11	99.8	99.4	98.9	96.1	89.4	74.3	57.6	31.4	1.3
1100–1199	12	99.5	99.1	98.1	95.1	89.0	72.4	57.2	34.6	1.4
1200–1299	14	99.8	99.7	99.4	97.2	91.4	77.2	61.8	37.6	1.6
1300–1399	3	99.8	99.7	99.6	98.7	93.6	80.6	68.3	41.3	2.0
1400–1499	7	100.0	100.0	99.8	98.8	94.7	81.6	68.2	44.2	1.7
>1500	13	100.0	100.0	99.9	99.2	96.5	84.8	72.4	51.3	2.2

Upon variant annotation (Fig 2), a total of 24,915 variants (2,858 unique) were called. After filtering for variants in the 10 genes (Table 1), a total of 1960 (287 unique) remained, 74 (56 unique) of which had MAFs less than 2% in both ethnicities (Table 3; Fig 2). A total of 61 of the 74 variants were validated and classified as TPs, averaging a sequencing depth of 659X and an allele balance of 0.51; this included 100% of the variants in seven out of the 10 genes (*ATM*, *BRCA2*, *CHEK2*, *NBN*, *PALB2*, *STK11*, and *TP53*), resulting in FDRs of 0 (Table 3). *BRCA1* and *CDH1* each had one FP, and *PTEN* had the highest FDR with 11 FPs, all in intron7/exon8 (Table 3). Despite that the average FP sequencing depth was 34X (ranging from 12X-63X), sequencing depths of the three regions harboring FPs revealed that all achieved an average greater than 427X (Table 3 and S3 Table). The average FP allele balance was 0.33, ranging from 0.13–0.68.

**Table 3:** Summary of called variants after bioinformatics pipeline and variant filtering.

Gene Name	Chr	Start position	Ref. Allele	Alt. Allele	Function	Exon/ Intron	DNA Change	Amino Acid Change	Polyphen2 prediction~	EA EVS^#^	AA EVS^#^	CLINVAR^	Number of individuals with variant called	Individual/ Sample	Ethnicity	GT	GQ	Total Depth	Allele balance	≥ 100X Depth AND 0.40 Allele Balance	Validation results (TP/FP)	Percent validated	FDR		Sensitivity	Specificity
																							Including all called variants	Excluding variants with ≤ 0.20 allele balance		
***ATM (NM_000051)***	chr11	108227849	C	G	NS	exon 3	c.146C>G	p.S49C	P	0.0136	0.0027	Risk Factor	1	1CAI-a	AA	Het	99	830	0.48	Yes	TP	100%	0.00	0.00	N/A	N/A
	chr11	108229171	C	T	Intronic	intron 3	c.186-7C>T	N/A	.	.	0.0142	Benign	1	4CA-a	AA	Het	99	1685	0.43	Yes	TP					
	chr11	108244860	C	T	NS	exon 7	c.735C>T	p.V245V	.	0.0134	0.0023	Likely Benign	1	1ED-a	EA	Het	99	907	0.50	Yes	TP					
	chr11	108248927	T	G	Intronic	intron 8	c.1066-6T>G	N/A	.	0.0026	0.0002	VUS	1	1CBE-a	EA	Het	99	79	0.43	No	TP					
	chr11	108249096	T	C	NS	exon 9	c.1229T>C	p.V410A	B	0.0022	0.0009	VUS	1	1EE-a	EA	Het	99	376	0.64	Yes	TP					
	chr11	108251973	T	C	NS	exon 11	c.1744T>C	p.F582L	B	0.0009	.	Likely Benign	1	1EAD-a	EA	Het	99	240	0.58	Yes	TP					
	chr11	108253901	T	C	S	exon 13	c.1986T>C	p.F662F	.	0.0006	.	Likely Benign	1	1EAC-a	EA	Het	99	331	0.54	Yes	TP					
	chr11	108254034	T	C	NS	exon 13	c.2119T>C	p.S707P	B	0.0109	0.0039	Likely Benign	1	1CBC-a	AA	Het	99	581	0.41	Yes	TP					
	chr11	108259051	C	A	NS	exon 16	c.2442C>A	p.D814E	B	0.0001	0.0198	Likely Benign	1	1EBA-a	AA	Het	99	195	0.37	No	TP					
	chr11	108267276	T	C	NS	exon 17	c.2572T>C	p.F858L	P	0.012	0.0032	Benign	1	1EAJ-a	EA	Het	99	227	0.47	Yes	TP					
	chr11	108284478	G	T	Intronic	intron 26	c.3993+5G>T	N/A	.	.	0.0098	Likely Benign	1	1CAD-f	AA	Het	99	368	0.59	Yes	TP					
	chr11	108315883	G	A	NS	exon 41	c.6067G>A	p.G2023R	D	0.0031	0.0007	VUS	1	1CG-a	EA	Het	99	1725	0.54	Yes	TP					
	chr11	108315904	A	G	NS	exon 41	c.6088A>G	p.I2030V	B	.	0.0148	Likely Benign	1	1CAF-a	AA	Het	99	745	0.43	Yes	TP					
	chr11	108317409	G	A	NS	exon 43	c.6235G>A	p.V2079I	B	0.0006	0.0166	Benign	1	1EA-a	EA	Het	99	997	0.51	Yes	TP					
	chr11	108327713	G	A	S	exon 48	c.7044G>A	p.T2348T	.	.	0.0014	Likely Benign	1	1CAC-a	AA	Het	99	103	0.59	Yes	TP					
***BRCA1 (NM_007300)***	chr17	43049113	A	G	Intronic	intron 22	c.5469+8T>C	N/A	.	.	0.0145	Likely Benign	2	3CC-a	AA	Het	99	1756	0.45	Yes	TP	91.67%	0.08	0.00	100%	92.3%
														4CA-a	AA	Het	99	1343	0.51	Yes	TP					
	chr17	43051071	A	C	NS	exon 21	c.5387T>G	p.M1796R	D	.	.	Pathogenic	1	1CAD-a	AA	Het	99	114	0.60	Yes	TP					
	chr17	43070958	C	T	NS	exon 16	c.5019G>A	p.M1673I	B	0.0152	0.002	VUS	1	1EA-a	EA	Het	99	457	0.49	Yes	TP					
	chr17	43091492	T	C	NS	exon 10	c.4039A>G	p.R1347G	B	0.0067	0.0011	VUS	1	1EAC-a	EA	Het	99	1579	0.49	Yes	TP					
	chr17	43092362	T	C	NS	exon 10	c.3169A>G	p.S1057G	B	.	.	VUS	1	1CE-a	AA	Het	99	131	0.51	Yes	TP					
	chr17	43092509	T	C	NS	exon 10	c.3022A>G	p.M1008V	B	.	0.0023	VUS	1	1EAG-a	AA	Het	99	187	0.42	Yes	TP					
	chr17	43093035	T	A	S	exon 10	c.2496A>T	p.P832P	.	.	.	Likely benign	1	1CD-a	AA	Het	99	1456	0.48	Yes	TP					
	chr17	43093626	A	G	S	exon 10	c.1905T>C	p.N635N	.	.	0.0005	Likely benign	1	1CF-a	AA	Het	99	264	0.52	Yes	TP					
	chr17	43094408	G	T	NS	exon 10	c.1123C>A	p.L375I	P	.	.	.	1	1CG-a	EA	Het	99	63	0.13	No	FP					
	chr17	43097280	G	T	NS	exon 7	c.557C>A	p.S186Y	D	.	0.0068	VUS	2	3CC-a	AA	Het	99	814	0.50	Yes	TP					
														4CA-a	AA	Het	99	407	0.50	Yes	TP					
***BRCA2 (NM_000059)***	chr13	32332629	C	T	NS	exon 10	c.1151C>T	p.S384F	D	0.0015	0.0002	VUS	1	1CD-a	AA	Het	99	1229	0.52	Yes	TP	100%	0.00	0.00		
	chr13	32332753	A	G	S	exon 10	c.1275A>G	p.E425E	.	.	0.0098	Benign	1	1CAI-a	AA	Homo	99	1046	1.00	Yes	TP					
	chr13	32333266	T	C	S	exon 10	c.1788T>C	p.D596D	.	0.0003	0.0163	Likely Benign	2	1CCB-a	AA	Het	99	1115	0.51	Yes	TP					
														1EB-a	AA	Het	99	347	0.50	Yes	TP					
	chr13	32333276	T	C	NS	exon 10	c.1798T>C	p.Y600H	B	.	0.0055	VUS	1	1CAG-a	AA	Het	99	72	0.42	No	TP					
	chr13	32333395	TG	-	Intronic	intron 10	c.1909+8delTG	N/A	.	0.0005	0.0059	VUS	1	1CAG-a	AA	Het	99	19	0.58	No	TP					
	chr13	32339375	A	G	NS	exon 11	c.5020A>G	p.S1674G	B	.	.	VUS	2	1CAD-a	AA	Het	99	344	0.61	Yes	TP					
														1CAD-f	AA	Het	99	1294	0.47	Yes	TP					
	chr13	32339554	C	T	S	exon 11	c.5199C>T	p.S1733S	.	0.0054	0.0009	Likely Benign	1	1CBE-a	EA	Het	99	1398	0.47	Yes	TP					
	chr13	32339966_ 32339970	AGTAA	-	FSD	exon 11	c.5611_5615 delAGTAA^$^	p.S1871fs^$^	.	.	.	Pathogenic	1	1CB-a	AA	Het	99	279	0.57	Yes	TP					
	chr13	32340678	G	A	NS	exon 11	c.6323G>A	p.R2108H	B	0.0015	0.0068	VUS	1	1CAH-a	AA	Homo	99	1,090	1.00	Yes	TP					
	chr13	32357750	G	A	S	exon 16	c.7626G>A	p.T2542T	.	.	0.0061	Likely Benign	1	1EAH-a	AA	Het	99	36	0.33	No	TP					
	chr13	32363385	T	C	NS	exon 18	c.8183T>C	p.V2728A	P	.	.	VUS	1	1CAD-f	AA	Het	99	1001	0.52	Yes	TP					
	chr13	32371035	A	C	NS	exon 20	c.8567A>C	p.E2856A	D	0.002	0.0005	VUS	1	1CAB-a	EA	Het	99	47	0.55	No	TP					
***CDH1 (NM_004360)***	chr16	68801830	A	G	S	exon 3	c.324A>G	p.R108R	.	0.0003	0.0061	Likely Benign	2	1CAF-a	AA	Het	99	1,210	0.49	Yes	TP	75%	0.25	0.00	N/A	N/A
														1CBH-a	AA	Het	99	120	0.48	Yes	TP					
	chr16	68813324	G	T	NS	exon 9	c.1149G>T	p.Q383H	B	.	.	.	1	1EE-a	EA	Het	99	20	0.20	No	FP					
	chr16	68819394	G	C	S	exon 11	c.1680G>C	p.T560T	.	0.0029	0.0007	Likely Benign	1	1CAI-a	AA	Het	99	3,053	0.53	Yes	TP					
***CHEK2 (NM_001005735)***	chr22	28695232	A	G	NS	exon 13	c.1399T>C	p.Y467H	D	0.0003	.	VUS	1	1EA-a	EA	Het	99	567	0.47	Yes	TP	100%	0.00	0.00	N/A	N/A
***NBN (NM_002485)***	chr8	89955458	T	C	NS	exon 10	c.1222A>G	p.K408E	P	.	0.0082	Likely Benign	1	1CC-a	AA	Het	99	1341	0.53	Yes	TP	100%	0.00	0.00	N/A	N/A
	chr8	89980833	A	G	S	exon 4	c.381T>C	p.A127A	.	0.0045	0.0018	Likely Benign	1	1EAJ-a	EA	Het	99	164	0.45	Yes	TP					
	chr8	89984520	C	T	Intronic	intron 1	c.37+5G>A	N/A	.	0.001	0.0182	Likely Benign	2	1CAH-a	AA	Het	99	177	0.54	Yes	TP					
														1CC-a	AA	Het	99	109	0.53	Yes	TP					
***PALB2 (NM_024675)***	chr16	23629898	T	C	S	exon 5	c.2256A>G	p.G752G	.	.	0.0055	Likely Benign	1	1CCB-a	AA	Het	99	1152	0.51	Yes	TP	100%	0.00	0.00	N/A	N/A
	chr16	23635127	T	G	S	exon 4	c.1419A>C	p.P473P	.	.	0.0084	Likely Benign	1	1CAI-a	AA	Het	99	955	0.49	Yes	TP					
	chr16	23635536	A	G	NS	exon 4	c.1010T>C	p.L337S	B	0.0197	0.0036	VUS	1	1EG-a	EA	Het	99	281	0.52	Yes	TP					
	chr16	23638125	T	C	NS	exon 2	c.53A>G	p.K18R	D	.	0.0155	VUS	2	1CC-a	AA	Het	99	276	0.49	Yes	TP					
														1EAG-a	AA	Het	99	111	0.42	Yes	TP					
***PTEN (NM_000314)***	chr10	87931070	C	T	S	exon 4	c.234C>T	p.T78T	.	.	0.0002	Likely Benign	1	1CA-a	EA	Het	99	339	0.53	Yes	TP	8.33%	0.92	0.92	N/A	N/A
	chr10	87960892	A	T	Intronic	intron 7	c.802-2A>T	N/A	.	.	.	Pathogenic	4	1CAF-a	AA	Het	91	27	0.30	No	FP					
														1CD-a	AA	Het	99	45	0.29	No	FP					
														1CF-a	AA	Het	96	41	0.46	No	FP					
														1EAJ-a	EA	Het	86	12	0.42	No	FP					
	chr10	87960896	C	A	NS	exon 8	c.804C>A	p.D268E	B	.	.	Not provided	3	1CC-a	AA	Het	99	32	0.34	No	FP					
														1EE-a	EA	Het	83	34	0.24	No	FP					
														3CC-a	AA	Het	99	63	0.27	No	FP					
	chr10	87960902	G	T	NS	exon 8	c.810G>T	p.M270I	P	.	.	.	3	1CAB-a	EA	Het	90	19	0.32	No	FP					
														1CAF-a	AA	Het	90	25	0.32	No	FP					
														1CC-a	AA	Het	41	34	0.68	No	FP					
	chr10	87960906	C	T	NS	exon 8	c.814C>T	p.H272Y	D	.	.	.	1	1CC-a	AA	Het	89	31	0.29	No	FP					
***STK11 (NM_000455)***	chr19	1218495	G	A	S	exon 2	c.369G>A	p.Q123Q	.	0.0002	0.0154	Likely Benign	5	1CAG-a	AA	Het	99	107	0.46	Yes	TP	100%	0.00	0.00	N/A	N/A
														1CBH-a	AA	Het	99	81	0.57	No	TP					
														1CE-a	AA	Het	99	88	0.49	No	TP					
														1EBA-a	AA	Het	99	849	0.51	Yes	TP					
														1EB-a	AA	Het	99	904	0.49	Yes	TP					
***TP53 (NM_000546)***	chr17	7670613	A	C	NS	exon 10	c.1096T>G	p.S366A	B	.	.	VUS	1	1EBA-a	AA	Het	99	863	0.43	Yes	TP	100%	0.00	0.00	N/A	N/A
	chr17	7673776	G	A	NS	exon 8	c.844C>T	p.R282W	D	0.0002	.	Pathogenic	1	1EC-a	AA	Het	99	240	0.28	No	TP					

Table 3 Key: (Chr) Chromosome; (Ref.) Reference; (Alt.) Alternate; (NS) Nonsynonymous; (S) Synonymous; (FSD) Frame-shift deletion; (VUS) Variant of Uncertain Significance; (EA) European American; (AA) African American

(~) Polyphen2 HDIV prediction; (B) Benign; (P) Probably damaging; (D) Damaging

(#) esp6500siv2

(^) most severe clinical significance classification; (GT) Genotype; (GQ) Genotype quality; (Het) Heterozygous; (Homo) Homozygous; (FDR) False discovery rate; (TP) True positive; (FP) False positive

($) The deletion was named using ANNOVAR (v.); however, it is within a short tandem repeat and commonly referred to as *BRCA2* c.5616_5620del5 (p.K1872Nfs) since Human Genome Variant Society (HGVS) nomenclature rules state to arbitrarily assign the deletion to the most 3’ nucleotide.

Though not optimal, low sequencing depth was not always indicative of a FP. Of the 20 variants covered less than 100X, 13 were FPs, and 7 were TPs with average sequencing depth of 60X and allele balance of 0.48, ranging from 0.33–0.58 (Table 3). In contrast, higher coverage, such as sequencing depth greater than 100X, was an indicator of a TP; all 54 variants covered over 100X were determined to be TPs. This included two homozygous TPs, which were each covered over 1000X with the alternate allele being the only one detected (Table 3), and 52 heterozygous TPs that had an average sequencing depth of 724X and allele balance of 0.50. To note the importance of allele balance, 95% of the TPs had an allele balance above 0.40 compared to only 23% of the FPs. Eighty five percent of the TPs had over 100X coverage and an allele balance of 0.40 (Table 3). No variants with an allele balance less than 0.20 were TPs, and additional filtering to exclude such variants improved FDRs (Table 3).

**Fig 3:**
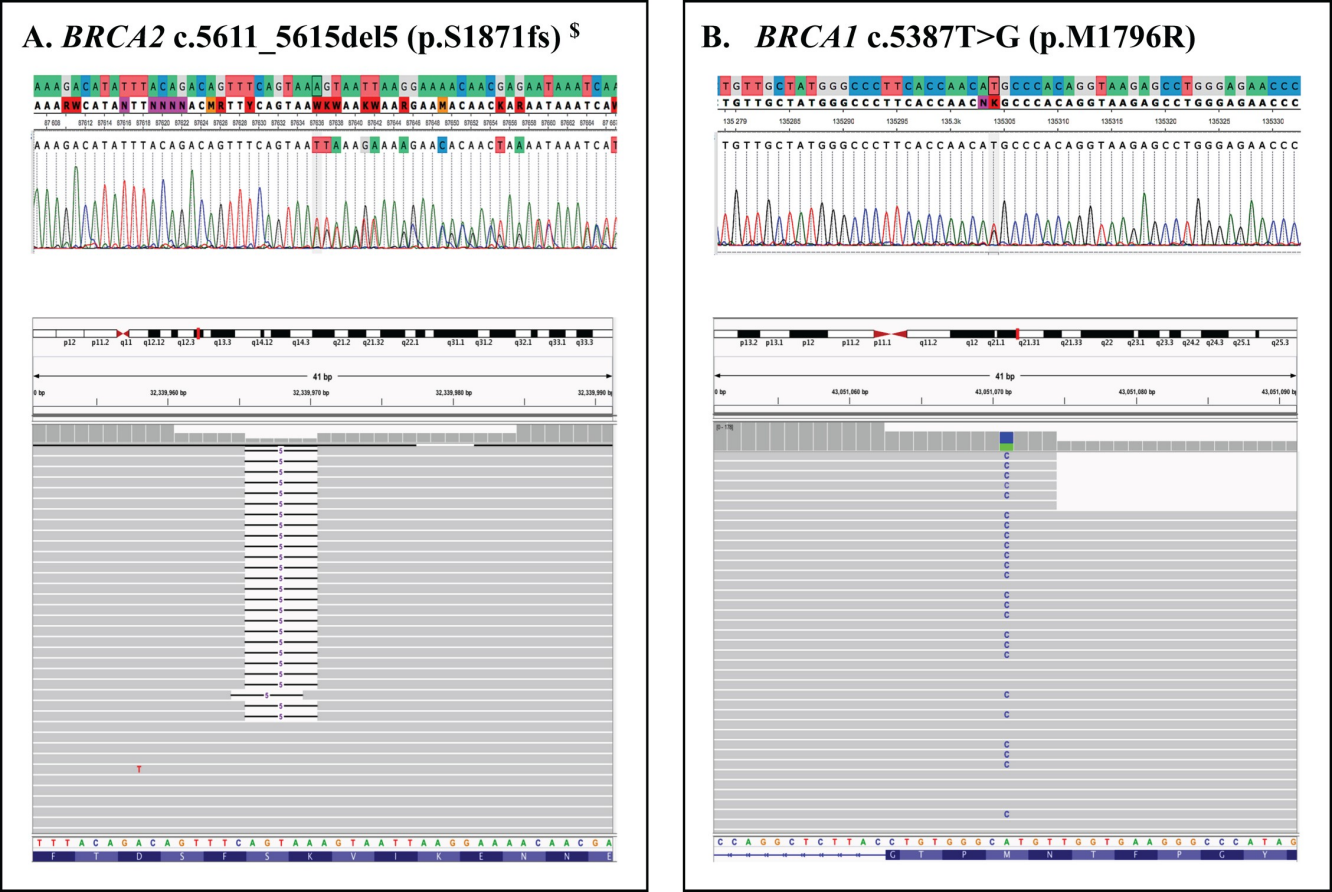
B.O.P. positive controls: Mutations that were previously reported through clinical gene screening. The Sanger sequence electropherogram (above) and the Integrative Genomics Viewer (v.2.4.5) image (below) are depicted for two B.O.P. screened positive controls: 1CB-a (Panel A) and 1CAD-a (Panel B). ($) This deletion is within a short tandem repeat and is commonly referred to as BRCA2 c.5616_5620del5 (p.K1872Nfs) since HGVS nomenclature rules arbitrarily assign the deletion to the most 3’ nucleotide.

Prior to B.O.P. screening, positive and negative *BRCA1* and *BRCA2* mutation status was known for eight of the 43 study participants; thus, sensitivity and specificity could be calculated for those genes. Seven study participants had previously undergone clinical *BRCA1/2* screening; six reported negative results with no pathogenic variants identified. One individual, 1CB-a, received a positive report indicating a pathogenic *BRCA2* frame-shifting mutation (c.5611_5615delAGTAA [p.S1871fs] also known as c.5616_5620delAGTAA [p.K1872Nfs), which was confirmed using PCR and Sanger sequencing prior to B.O.P. screening (Fig 3 and Table 3). The eighth individual, 1CAD-a, had not personally obtained clinical gene screening; however, a deceased family member had undergone clinical *BRCA1/2* screening and received a positive report indicating a pathogenic missense mutation (*BRCA1* c.5387T>G [p.M1796R]). Thus, this individual was screened for the familial mutation using PCR and Sanger sequencing prior to B.O.P. screening and tested positive (Fig 3 and Table 3). Noteworthy, another BC-affected family member, 1CAD-f, tested negative for *BRCA1* p.M1796R in the research laboratory prior to B.O.P. screening but could not be considered a TN for the specificity calculation since full gene screening had not been carried out (Table 3). B.O.P. variant calling reported 12 and 14 variants in *BRCA1* and *BRCA2*, respectively (Table 3). Upon Sanger sequencing confirmation, this included 11 TPs, zero FNs, six TNs and one FP in *BRCA1*, and 14 TPs, six TNs, and zero FNs and FPs in *BRCA2* (Table 3), which corroborated the previously reported *BRCA1* and *BRCA2* mutation statuses. Therefore, B.O.P. screening of *BRCA1/2* resulted in 100% sensitivity and 92.3% specificity. However, specificity became 100% with the elimination of variants with an allele balance of 0.20 or less.

Of the 61 TPs, 45 were detected in AAs; this included 34 unique variants, eight of which were detected in multiple individuals (Table 3). According to ClinVar [13, 14], the 34 variants were categorized as pathogenic/risk factor (n = 4), VUSs (n = 11), or benign/likely benign (n = 19). A total of five variants were predicted to be deleterious in Polyphen, two of which have been defined as pathogenic non-synonymous variants in ClinVar; the other three are currently classified as VUSs (Table 3). Of the eight variants detected in more than one individual, *BRCA2* c.5020A>G; p.S1674G, was identified in two first cousins. The remaining seven were in seemingly unrelated individuals. This includes *STK11* c.369G>A;p.Q123Q, a seemingly benign variant, which is reported to have a MAF of 1.5% in the general AA population but was detected in five of the 23 AAs in this study, indicating a MAF of 10.8% (*P value* 8.50 X 10^−4^; *Odds ratio* 7.79 CI_95_[2.32–20.70]). Furthermore, the 45 AA TPs were detected in 96% of the AAs screened, and multiple variants were detected in 70% of the cases. In contrast, 16 TPs were validated in 55% of the EAs, and only 20% had multiple variants. The difference in the number of individuals from each ethnicity with at least one TP was significant (*P value* 2.71 X 10^−3^; *Odds ratio* 16.83 CI_95_[1.93–819.72]) as well as the number of cases from each ethnicity with multiple TPs (*P value* 1.95 X 10^−3^; *Odds ratio* 8.60 CI_95_[1.89–49.30]). No overtly pathogenic variants were validated in EAs, but 50% of the EA TPs (8/16) were listed as a VUS, three of which were predicted to be deleterious in Polyphen [15].

## Discussion

Our group has developed B.O.P., a research-based NGS gene panel, which targets 87 genes that have been suggested, predicted, or clinically proven to be associated with risk of BC, OvC, and/or PC. The overall purpose of this new panel is to gain additional insights toward the genetic risk of and overlap between those three cancers. This manuscript served to introduce B.O.P. by reporting its initial screening, which involved 43 cancer-affected individuals from the AHCC [16]. Targeting ~500 Kb of DNA, 98.9% of the base pairs were covered at least 1X, and an average sequencing depth of 809X was obtained. We took a closer look at 10 NCCN regulated genes in order to begin the analytical assessment of the panel and ensure the accurate detection of clinically relevant variants; upon variant filtering and validation, 100% of the variants in seven of the 10 genes were TPs. TPs had an average sequencing depth of 659X and allele balance of 0.51, whereas the average FP sequencing depth and allele balance was 34X and 0.33, respectively. Although FPs had a much lower average sequencing depth compared to TPs, a low sequencing depth was not always indicative of a FP. Contrarily, all variants called with high sequencing depths (>100X) signified a TPs. Furthermore, sensitivity and specificity of *BRCA1/2* were calculated to be 100% and 92.3%, respectively.

There are a number of different targeted enrichment options to choose from when designing gene panels, all of which can have different affects on sequencing outcomes [10]. When comparing methods, Samorodnitsky *et al*. concluded that Haloplex had the highest on-target read alignment and normalized sequencing depth but the least uniformity [10]. Noteworthy, despite reports of Haloplex resulting in a high percentage (>90%) of on-target read alignments [10, 17], 50.9% of our QC passed reads mapped to the B.O.P. targeted regions. With other Haloplex gene panel studies not reporting such data [18–20], it is difficult to make general conclusions about Haloplex on-target read alignment specificity. However, similar on-target read alignment percentages have been reported; Castera *et al*. used SureSelect baits in order to target hereditary BC and OvC susceptibility genes, and reported an average of 42% of reads on-target [21]. Ultimately, the percentage of off-target reads is likely dependent on a number of factors, including the specific genes/regions being targeted [22]. Of the reads that mapped on-target, B.O.P.’s overall sequencing depth averaged 809X, and each individually assessed gene obtained average sequencing depths from 505X-1017X. Nevertheless, large interquartile ranges indicated that depth was not uniform. This was expected since no current enrichment and sequencing approach provides complete uniformity primarily because of complex genomic regions that are very difficult to capture/sequence and result in low sequencing depths or even no coverage at all [10, 22].

By focusing on a select set of genes/regions, NGS gene panels target a smaller number of base pairs compared to more broad applications such as exome and whole genome sequencing. The smaller target-capture provides the option to achieve a high average sequencing depth, which aids in variant identification [10, 23]. Therefore, the overall goal is to obtain 100% coverage as well as the appropriate/desired sequencing depths at all targeted base pairs. Since this goal is not generally achieved, complementary assays can be used to fill in gaps, which is commonly implemented for clinical applications. In such cases, regions of low/no coverage are normally Sanger sequenced [22, 23]. B.O.P. was able to cover, on average, 98.2% of its targeted base pairs at 1X. Being a research panel, no gap-filling assays were carried out; however, region-specific coverage analyses provided insight towards the feasibility of gap-filling. Gap-filling criteria has been described in a number of BC NGS gene panel publications, specifically, those that highlighted panel performance and analytical validity [21, 24–26]. Being clinical panels, regions covered less than 20X [21] or 50X [24–26] were checked by conventional methods. Interestingly, only two B.O.P. regions had average sequencing depths less than 100X (68X and 82X), which would not have required complementary assays to fill in gaps according to the criteria set in the referenced studies [21, 24–26]. This is despite that, on average, those two B.O.P. regions missed 24.3% of the targeted base pairs and only covered 52.2%, 34.6%, and 21.3% of targeted base pairs at or greater than 20X, 50X, and 100X, respectively. Furthermore, 63 of the 225 B.O.P. regions-of-focus did not, on average, obtain 100% coverage at 1X; these regions had average sequencing depths ranging from 68X-1354X. Thus, regions with high sequencing depths still had base pairs with no/low coverage, which happened to be where FPs were detected in this study. Therefore, only gap-filling regions with ‘low’ (20X or 50X) sequencing depths, will not guarantee 100% coverage. Establishing mapping criteria to ensure all base pairs are covered at a desired depth is ideal but would likely reveal gaps in too many regions, making gap-filling infeasible. Overall, gaps in B.O.P. as well as other panels, even with gap-filling criteria, can provide less than definitive negative results [23]; however, in noting that, zero FNs were identified in *BRCA1* and *BRCA2*, resulting in 100% sensitivity.

In addition to gap-filling, conventional approaches are also used to validate called variants. A total of 1960 variants were detected in the 10 B.O.P. assessed genes and, to reduce the number of variants to validate for this analytical assessment, only variants with MAFs less than 2% in both ethnicities were Sanger sequenced. This included 74 variants, 61 of which were confirmed and defined as TPs revealing 13 FPs. The validation process ultimately provided insight regarding the likelihood of confirmation based on variant quality, such as sequencing depth since all 54 variants covered over 100X were TPs. These results corroborated the criteria established by Mu *et al*., which set high confidence calls as having a minimum sequencing depth of 100X and allele balance of 40%. Additionally, Mu *et al*. indicated that such calls did not require confirmation. Although, all B.O.P. variants covered at or above 100X were TPs despite allele balance, the criteria from Mu *et al*. will be implemented in the future in order to be thorough. This will limit validation efforts to low confidence calls, reducing the cost and time of validation.

In this study, 22 variants had low confidence calls. This included nine TPs, seven of which were covered less than 100X and two that failed to meet the required allele balance. The remaining 13 were FPs with an average sequencing depth of 34X and allele balance of 0.33, reiterating that low sequencing depths are susceptible to sequencing artifacts [10, 23]. Interestingly, as mentioned in the previous paragraph, the regions harboring the FPs did not have low sequencing depths, stressing the potential lack of uniformity within a targeted region. On another note, 11 of the 13 FPs were in *PTEN*. Considering *PTEN* has a processed pseudogene, *PTENP1* on chromosome 9, their homology could have contributed to probe mis-priming as well as read mis-alignments. Overall, encountering problematic regions, such as regions with high homology or GC rich content, is common and referred to in many studies [18, 22, 23]. Overall, for each assessed gene, FDRs ranged from 0 to 0.92, the latter being *PTEN*. Of course, FDRs improved as additional filtering was implemented. Initial B.O.P. specificity, which could only be calculated for *BRCA1*/*BRCA2*, was 92.3%. Upon filtering out variants with an allele balance equal to or less than 0.20, specificity was 100%. Ultimately, Sanger sequencing all low confidence calls will eliminate FPs and provide 100% specificity; therefore, it is common to complement NGS gene panels with Sanger sequencing validation in order to consider the test complete and optimize specificity [21, 24–28].

In addition to enabling B.O.P.’s initial analytical assessment, the first B.O.P. screening, which involved 23 AAs and 20 EAs from the AHCC [9], has provided insight regarding variant contributions and ethnic differences. Overall, compared to EAs, AAs had a significantly higher number of individuals with at least one TP (*P value* 2.71 X 10^−3^) as well as individuals with multiple TPs (*P value* 1.95 X 10^−3^). Of course, comparisons to ethnic-specific controls will determine if these differences contribute to an inherited cancer risk. Interestingly, according to ClinVar [13, 14], none of the variants identified in EAs were considered pathogenic/risk variants, whereas 17.4% (4/23) of the AAs had a variant with that classification. The majority of the detected variants were classified as VUSs or benign/likely benign; ultimately, elucidating how VUSs and, even, synonymous variants contribute towards risk is very important. Synonymous variants, though normally ignored and considered benign, can affect splicing, gene expression or translation dynamics, all of which can contribute to a disease phenotype [29]. To further stress their importance, they have been reported to act as driver mutations in human cancers [30] and, through this initial B.O.P. screening, *STK11* c.369G>A;p.Q123Q was detected in significantly more AA cases than controls (*P value* 8.50 X 10^−4^). Additionally, despite recognizing that hereditary BC risk is polygenic [31], little effort has been put forth to thoroughly investigate all variants in clinically relevant BC susceptibility genes, not to mention variants in genes currently lacking clinical significance, and determine if different variant combinations increase risk. Altogether, seemingly benign variant combinations could, in fact, be pathogenic, and paired with the striking difference between ethnicities regarding the number of cases with multiple variants, further investigation is warranted. Of course, a larger number of cases will be required for this effort.

In summary, this effort assesses the analytical validity of the B.O.P. panel and demonstrates the panel’s ability to accurately detect mutations in 10 NCCN clinically actionable genes [8]. Despite the potential biases of the B.O.P. capture and NGS, the high depth of coverage, low FDR, and great sensitivity and specificity strongly support the use of this research gene panel to further elucidate hereditary BC/OvC/PC genetics. Although the cohort for this initial assessment is small, B.O.P. has begun to determine the mutation contributions of clinically valid genes in different ethnicities as well as permit the investigation of VUSs and other variant types and their effect towards polygenic risk. Furthermore, continued B.O.P. screening can provide additional evidence to confirm or refute previously suggested susceptibility genes, lessening the number of genes that lack clinical validity on commercially available panels [1]. Additionally, with the incorporation of candidate genes on B.O.P., it has the potential to identify novel genetic risk factors that are contributing towards BC, OvC, and PC. Lastly, as new susceptibility genes are discovered that are not currently on the B.O.P. panel, it is important to stress the ability to edit the targeted genes in order to best reflect clinical screening. As described herein, there are many potential benefits of B.O.P. screening, and we aim to make advances in cancer genetics research through its implementation in our research efforts.

## Supporting information

S1 Table Targeted genes on the B.O.P. panel. (XLSX)

S2 Table Percentage of mapped reads. (XLSX)

S3 Table Targeted regions of the 10 assessed genes and coverage. (XLSX)
